# The mHealth App Usability Questionnaire (MAUQ): Development and Validation Study

**DOI:** 10.2196/11500

**Published:** 2019-04-11

**Authors:** Leming Zhou, Jie Bao, I Made Agus Setiawan, Andi Saptono, Bambang Parmanto

**Affiliations:** 1 Department of Health Information Management University of Pittsburgh Pittsburgh, PA United States; 2 Department of Computer Science Udayana University Badung, Bali Indonesia

**Keywords:** questionnaire design, reliability and validity, mobile apps

## Abstract

**Background:**

After a mobile health (mHealth) app is created, an important step is to evaluate the usability of the app before it is released to the public. There are multiple ways of conducting a usability study, one of which is collecting target users’ feedback with a usability questionnaire. Different groups have used different questionnaires for mHealth app usability evaluation: The commonly used questionnaires are the System Usability Scale (SUS) and Post-Study System Usability Questionnaire (PSSUQ). However, the SUS and PSSUQ were not designed to evaluate the usability of mHealth apps. Self-written questionnaires are also commonly used for evaluation of mHealth app usability but they have not been validated.

**Objective:**

The goal of this project was to develop and validate a new mHealth app usability questionnaire.

**Methods:**

An mHealth app usability questionnaire (MAUQ) was designed by the research team based on a number of existing questionnaires used in previous mobile app usability studies, especially the well-validated questionnaires. MAUQ, SUS, and PSSUQ were then used to evaluate the usability of two mHealth apps: an interactive mHealth app and a standalone mHealth app. The reliability and validity of the new questionnaire were evaluated. The correlation coefficients among MAUQ, SUS, and PSSUQ were calculated.

**Results:**

In this study, 128 study participants provided responses to the questionnaire statements. Psychometric analysis indicated that the MAUQ has three subscales and their internal consistency reliability is high. The relevant subscales correlated well with the subscales of the PSSUQ. The overall scale also strongly correlated with the PSSUQ and SUS. Four versions of the MAUQ were created in relation to the type of app (interactive or standalone) and target user of the app (patient or provider). A website has been created to make it convenient for mHealth app developers to use this new questionnaire in order to assess the usability of their mHealth apps.

**Conclusions:**

The newly created mHealth app usability questionnaire—MAUQ—has the reliability and validity required to assess mHealth app usability.

## Introduction

### Background

Mobile health (mHealth) apps can be used to perform tasks in areas such as wellness management, behavior change, health data collection, disease management, self-diagnosis, and rehabilitation as well as act as an electronic patient portal and medication reminder [1,2]. A number of research studies have been performed on mHealth apps, and the results have indicated that well-designed mHealth apps can empower patients, improve medication adherence, and reduce the cost of health care [3-6]. However, a previous study pointed out that roughly half of mHealth app users stop using some mHealth apps for various reasons such as hidden costs, high data-entry burden, and loss of interest [7]. Among these factors, high data-entry burden is clearly a usability issue, while loss of interest may also be triggered by poor usability of an app. These facts indicate the importance of good usability of mobile apps.

Among several methods for evaluation of mobile app usability, the usability questionnaire is the most frequently used because of its simplicity in terms of execution and data analysis. Ironically, although millions of mobile apps have been created and released to the public in the past decade [8], *no highly reliable usability questionnaire has been specifically designed for mobile apps.*

There have been studies on creating new mobile app *usability models* because of the special components in mobile apps such as small form factors, connectivity issues, battery issues, limited computation power, and the unique security and privacy challenges associated with highly portal mobile devices [9,10].

Studies have also tried to adjust existing usability frameworks and questionnaires, so that the models can be used for evaluation of mobile app usability [11,12]. However, in practice, many authors of the published questionnaire-based mobile app usability studies chose one of the following two options: use of well-validated usability questionnaires designed for general software systems or creation of their own usability questionnaire according to the general guidelines of usability assessment.

Both choices have their advantages and disadvantages. The advantage of the former case is that the questionnaires have been used in many other studies, and therefore, certain usability aspects of the mobile app can be reliably measured. Frequently used usability questionnaires are the System Usability Scale (SUS) and Post-Study System Usability Questionnaire (PSSUQ) [13,14]. However, for the aspects that are unique to mobile apps, these questionnaires cannot provide the desired unique information in mobile apps.

In the second case, when authors create their own usability questionnaire, they have the flexibility to cover the unique features offered by their mobile apps; however, because the major focus of those usability studies was not on validation of their self-written questionnaires, the authors typically only recruited a small number of study participants; therefore, the obtained data were not sufficient for a reliable psychometric analysis. In other words, the reliability and validity of results from the latter usability studies are questionable. Therefore, there is a need to create a reliable usability questionnaire specifically for mobile apps. This study will specifically focus on creating an mHealth app usability questionnaire.

### Requirements for a New Usability Questionnaire

When designing a user-based mHealth app usability questionnaire, one has to consider the type of users and type of mobile apps.

All mHealth apps can be arranged into one of two categories according to the type of target users: patients or health care providers. The type of user is not determined by the occupation of the user, but rather by the *purpose* for using the app. Here, patients are people who use an mHealth app to maintain, improve, or manage their own health, while health care providers are people who use an mHealth app to *deliver* health care services such as medication prescription, laboratory ordering, consultation, and patient education. In the usability questionnaire, certain words need to be customizable to reflect different target users of mHealth apps.

The mHealth apps can be further grouped according to the nature of the *interaction* between the patients and health care providers in the app: *interactive* mHealth apps and *standalone* mHealth apps. In interactive mHealth apps, the app users can send and receive information from their health care providers or patients via the app. The communication between patients and providers can be synchronous or asynchronous. In standalone mHealth apps, the app users enter/collect/store health information about themselves or other people. The standalone apps may generate reminders or show a summary or details about the collected health information, but these apps do not send the data to the user’s health care providers or patients. In other words, the major difference between these two types of apps is the level of interactivity. For interactive mHealth apps, there is direct interaction between patients and their health care providers. Therefore, the questionnaire for interactive apps should have statements about the quality of interaction, while the questionnaire for standalone apps may not need those statements.

Hence, *four different versions* of the mHealth app usability questionnaire are needed to evaluate the usability of mHealth apps designed for different users (patients or health care providers) and different interaction modes (interactive or standalone).

### Objectives

In this study, the goal was to create a short, reliable, and customizable (via four different versions) questionnaire for assessing the usability of mHealth apps.

## Methods

### Process Overview

To create the desired questionnaire, we reviewed a large number of published papers about mHealth apps and then created an mHealth app usability questionnaire (MAUQ) based on the questionnaires used in these published studies, taking into consideration the uniqueness of mobile devices and mHealth apps. We then used this newly developed questionnaire in a usability study on two mHealth apps. A psychometric analysis was performed to evaluate the reliability of the MAUQ as well as the correlation between the results from the MAUQ and those from the SUS and PSSUQ.

### Usability Questionnaire Development

#### Step 1: Collecting Usability Questionnaire Statements From Existing Usability Questionnaires Used in mHealth App Studies

To design the desired mHealth app usability questionnaire, we first used the keywords “mobile app” and “usability” to search for published usability studies on mobile apps in PubMed, CINAHL (Cumulative Index to Nursing & Allied Health Literature), IEEE Xplore, ACM Digital Library, and InSpec [15]. From the 1271 articles obtained, we identified 125 questionnaire-based mHealth app usability studies and collected 38 individual questionnaires including well-validated questionnaires such as the SUS, PSSUQ, After Scenario Questionnaire [16], Perceived Usefulness and Ease of Use [17], Usefulness, Satisfaction, and Ease of use [18], Software Usability Measurement Inventory [19], Questionnaire for User Interaction Satisfaction [20], Computer Usability Satisfaction Questionnaire [16], Health IT Usability Evaluation Scale (Health ITUES) [21,22], and NASA Task Load Index [23] as well as a number of self-written usability questionnaires.

#### Step 2: Creating a Draft of the New mHealth App Usability Questionnaire and Assessing Each Statement’s Relevance and Clarity

We collected 312 *unique* questionnaire statements (including similar statements with different wording) from these 38 questionnaires and arranged them into categories according to the general guidelines for usability assessment and the unique features of mobile apps. The categories included items such as usefulness, ease of learning, ease of use, effectiveness, satisfaction, interface quality, interaction quality, reliability, error messages and online support, internet connectivity, and social settings of mobile app use [9,24-26]. These 312 statements were preprocessed by removing Yes/No questions, negatively phrased questions, and redundant questions. The remaining 140 statements were then distributed to seven researchers with extensive experience in mobile app usability studies (referred to as “usability experts” in the following descriptions) to collect their feedback on the *relevance* and *clarity* of these statements on a scale of 1-4, where 1 indicates no relevance or clarity and 4 indicates high relevance or clarity. If four or more usability experts rated the relevance of a statement 1 or 2, it was removed from the questionnaire. If any one of the usability experts rated the clarity of a statement 1 or 2, the wording of the statement was adjusted. After this step, 53 statements remained in the questionnaire, 19 of which needed the wording to be adjusted.

#### Step 3: Conducting Further Refinement on the Draft Questionnaire

After the necessary adjustments to the 19 statements, the research team held several face-to-face meetings with the seven usability experts to extensively discuss each of the 53 statements remaining. At the end of the discussion, the entire research team decided to reduce the number of statements for interactive mHealth apps to 21 and the number of statements for standalone mHealth apps to 18.

#### Step 4: Performing Usability Studies and Psychometric Analysis

**Figure 1:**
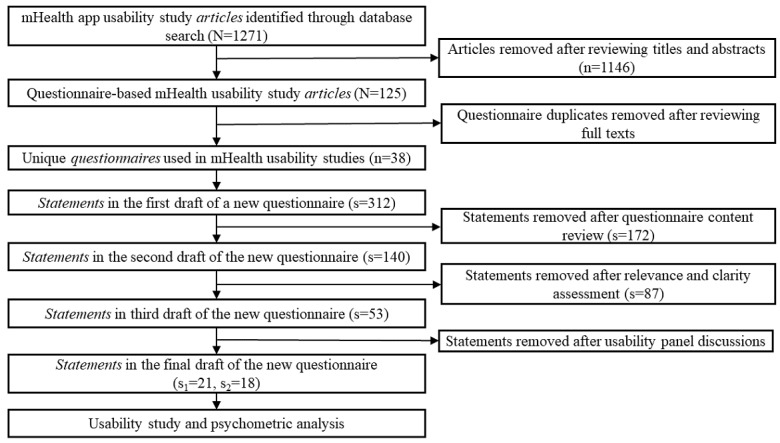
A flow chart of the new usability questionnaire development and validation. mHealth: mobile health; s: number of statements; s1: number of statements in the questionnaire for interactive mobile apps; s2: number of statements in the questionnaire for standalone mobile apps.

In this step, we recruited a group of study participants to take part in usability studies using this new questionnaire (MAUQ) and two commonly used usability questionnaires (PSSUQ and SUS). The data obtained from this study were used to evaluate the reliability and validity of this new questionnaire. Figure 1 visually depicts each specific step in the development and validation of the new usability questionnaire. Details of the usability study and the psychometric analysis are presented in the following sections.

### Study Design and Setting

After the new usability questionnaire was ready, a usability study on mobile apps was designed using the newly developed MAUQ and two widely used usability questionnaires, PSSUQ and SUS. Each of the study participants was asked to first use two mHealth apps: one was an interactive mobile app—iMHere 2.0 [3,27,28]—and the other was a standalone mobile app—Fitbit app version 2.36 on a 10-inch 32 GB iPad Air 2 (iOS version 10.3.2).

During the study, a general introduction to the purpose of the study and the two mHealth apps (iMHere 2.0 and Fitbit) was provided, along with a brief demo of these two mHealth apps and an explanation of the new usability questionnaire MAUQ. After the introduction, the study participants were asked to finish several tasks using these two mobile apps and provide responses to the three usability questionnaires (MAUQ, PSSUQ, and SUS) through the Web-based Qualtrics survey system (Qualtrics, Provo, UT). The demographic information of the study participants was also collected during this usability study via the Qualtrics system. Statistical analysis was performed on the collected data. This study protocol was approved by the institutional review board office at the University of Pittsburgh. A brief description about iMHere 2.0 and Fitbit apps and the tasks completed by the study participants in the usability study are provided below.

#### Apps Used in the Usability Study

The iMHere 2.0 system has multiple major components, including a mobile app for patients and a Web portal for clinicians. Patients can use the mobile app to perform a number of self-care tasks such as managing their medication schedules, reporting minor skin issues, performing mental health self-assessment, tracking physical activities and nutrition, sending messages to clinicians, receiving messages from clinicians, and receiving brief education in health-related topics. Clinicians can use the Web portal to view all the entered patient information and communicate with patients. Clinicians can also issue or change interventions for patients via the Web portal, which are reflected on the mobile app for patients in real time. Therefore, this mobile app in iMHere 2.0 is considered an interactive mHealth app.

The Fitbit app accompanies the Fitbit wearable device. It can display the data collected by the wearable device, such as the number of steps, heart rate, and sleep duration. The user can also enter some data that cannot be collected by the device, such as food and drink consumed in a day. Although the Fitbit app has some social media features, the user cannot directly communicate with his/her health care provider via the app. Therefore, this Fitbit app is considered a standalone mHealth app.

#### Tasks Performed by Subjects in the Usability Study

When using the iMHere app, study participants were asked to finish four tasks: (1) schedule a reminder for taking medication in the MyMeds module of the app; (2) report a skin problem in the Skincare module; (3) add medical history records such as medication history, allergies, immunizations, social history, and family medical history in the Personal Health Record module; and (4) adjust settings of the app, such as making the font bigger or changing the personal profile picture. The app would generate a reminder according to the indicated time point in the medication reminder schedule, and the study participant was required to respond to the reminder. After the study participants reported one skin problem, the data would show up on a Web-based clinician portal, a clinician provided a response to the subject, and the information was shown in the message section of the iMHere 2.0 app. Study participants were required to read the message and could choose to reply to the clinician, if necessary.

When using the Fitbit app, study participants were asked to finish the following tasks: determine the numbers of steps and distances walked in the past week and past month; check their sleep history and add one new sleep log; add one new record about food eaten that day; add one new record about beverages they had drunk that day; check messages received in the app; and change the setting of goals such as the desired number of steps, number of sleep hours, and current and desired weight.

### Study Participants

Participants were recruited through flyers distributed in the Greater Pittsburgh area and at the Pitt + Me website at the University of Pittsburgh [29]. Participants were screened using the following selection criteria: ability to speak fluent English; high school or higher education; age between 18 and 65 years; ability to communicate with others orally and in writing; and at least a few years of experience using smart devices such as a smartphone, tablet, or smartwatch. People who meet these selection criteria are the majority of smart device owners and mobile app users [30], and therefore, they are good candidates for providing valuable information in this usability study. All data of the eligible candidates were stored in one Excel file, and the actual study participants were randomly selected from this list.

### Statistical Methods

#### Descriptive Analysis

##### Data Preprocessing

Responses to the statements on the MAUQ and PSSUQ ranged from 1 (strongly agree) to 7 (strongly disagree). The responses to the statements on the SUS ranged from 1 (strongly disagree) to 5 (strongly agree). Nine (of 128, 7.0%) study participants missed one to a few statements, and the other 119 (of 128, 93.0%) study participants provided responses to all the statements on the MAUQ. In our analysis, we used a value of 4 (indicating neither agreement nor disagreement) to fill the position of the missing data for the MAUQ.

##### Score Conversion and Descriptive Statistics

A descriptive analysis was first performed on the collected data to gain an understanding of the demographic characteristics of the study participants and the overall performance of the three usability questionnaires (MAUQ, PSSUQ, and SUS). The means and SD for individual statements and the entire questionnaire for the MAUQ and PSSUQ were calculated. The PSSUQ version 3 used in this study had 16 items, where items 1-6 were the first subscale (PSSUQ1), items 7-12 were the second subscale (PSSUQ2), and items 13-15 were the third subscale (PSSUQ3) [14,31]. The scores for these three subscales and the total score for the entire scale for PSSUQ were calculated. For the SUS, we used the standard score conversion procedure to convert each study participant’s answers to one score between 0 and 100 [31].

##### Correlation Coefficient Calculation

The correlation coefficients among the scores obtained using the MAUQ, PSSUQ, and SUS were calculated, including the correlation coefficients of their subscales, if applicable, and the intersubscale correlation coefficient within the MAUQ. The former correlation coefficients were to be used to determine the criterion validity of the MAUQ, while the latter were to be used to determine the construct validity of the MAUQ [31]. In the criterion validity evaluation, a correlation coefficient as low as 0.30 or 0.40 is sufficient. In the construct validity evaluation, a low correlation is good for divergent validity and a high correlation is good for convergent validity.

#### Psychometric Analysis

An exploratory factor analysis (EFA) was performed on the data collected from all study participants using the MAUQ. We expected multiple factors for the MAUQ and that these factors would not be totally independent. Therefore, we used maximum likelihood as the method for factor extraction and quartimin with Kaiser Normalization for oblique rotation in EFA [32]. The factor loadings obtained in the EFA were used to determine whether each item should be included in the usability questionnaire and one specific construct. Here, 0.32 was used as a guiding value for the evaluation [33]. However, in certain cases, we overruled this value and chose to keep a statement in the questionnaire, even if the factor loadings were smaller than 0.32 or multiple factor loadings were greater than 0.32, using judgement skills gained from our extensive experience in mHealth app usability studies.

To evaluate the internal consistency of the MAUQ, we calculated the values of Cronbach alpha for the entire questionnaire and its subscales. Cronbach alpha is a commonly used measurement of internal consistency for questionnaires. For research and exploratory studies, Cronbach alpha values of 0.7-0.8 are acceptable, while a value of around 0.9 is excellent [34].

All these statistical analyses were performed using R 3.3 (R Foundation for Statistical Computing, Vienna, Austria) and IBM SPSS, version 24 (IBM Corp, Armonk, NY).

## Results

### Participants

In total, 128 participants were recruited from the Greater Pittsburgh area for the study. The demographic information of these participants is summarized in Table 1.

**Table 1:** Demographic characteristics of the study participants (N=128).

Characteristic		Value
**Age (years), mean (SD)**		**32.6 (13.12)**
	18-28	67 (52.3)
	29-50	42 (32.8)
	51-65	19 (14.8)
**Gender, n (%)**		
	Male	49 (38.3)
	Female	79 (61.7)
**Race, n (%)**		
	African American	22 (17.2)
	White	81 (63.3)
	Asian	22 (17.2)
	Other	3 (2.3)
**Education, n (%)**		
	High school diploma	5 (3.9)
	Some college credits, no degree	28 (21.9)
	Associate degree	9 (7.0)
	Bachelor’s degree	45 (35.2)
	Master’s degree	31 (24.2)
	Professional degree	5 (3.9)
	Doctoral degree	5 (3.9)
**Marital status, n (%)**		
	Single	87 (68.0)
	Married or long-term committed relationship	34 (26.6)
	Divorced or separated	7 (5.5)
**Living place, n (%)**		
	Urban	83 (64.8)
	Suburban	39 (30.5)
	Rural	6 (4.7)
**Employment, n (%)**		
	Employed	92 (71.9)
	Not employed	29 (21.9)
	Retired or Disabled	8 (6.2)
**Household income (US $ per annum), n (%)**		
	<10,000	24 (18.8)
	10,000-50,000	52 (40.6)
	50,001-100,000	27 (21.1)
	>100,000	17 (13.3)
	Decline to answer	8 (6.3)
**Occupation, n (%)**		
	Student	40 (31.3)
	Researcher	19 (14.8)
	Administrative Personnel	18 (14.1)
	Customer Service Personnel	10 (7.8)
	Other	39 (30.5)
Years of Using Mobile Devices, mean (SD)		6.86 (2.34)
Year of Using Mobile Apps, mean (SD)		6.64 (2.28)

### Descriptive Data

The distribution of data from the MAUQ-based usability study was not normal according to both the Shapiro-Wilk test and the Kolmogorov-Smirnov test, where *P*<.01. Therefore, when evaluating the impact of demographic characteristics on the answers in the MAUQ, we used a nonparametric Kruskal-Wallis test. The test results indicated that none of the demographic factors (age, gender, race, education, income, marital status, living place, occupation, employment status, and experience using smart device and mobile apps) had a statistically significant impact on the answers to the individual statements or the overall score on the MAUQ (*P*>.05 in all cases).

### Psychometric Analysis Results

#### mHealth App Usability Questionnaire for Interactive mHealth Apps (Patient Version)

Table 2 shows the factor loadings for the 21-item MAUQ designed for interactive mHealth apps. Evidently, there are three factors for the MAUQ when 0.32 is used as the cut-off value for factor loadings, with one exception—all three factor loadings for the ease of learning statement (“ *It was easy for me to learn to use the app”*) were smaller than 0.32; however, according to our experience working with usability studies and evidence from numerous other previous studies, we believe this statement is very important for a mobile app usability study and is closely related to the ease of use statement and the design of the interface. Therefore, we decided to retain this statement in the first construct of the questionnaire.

**Table 2:** Exploratory factor analysis results for the 21 items on the mHealth App Usability Questionnaire designed for interactive mHealth apps (overall Cronbach alpha=0.932, 21 items). Values >0.32 for each factor are italicized.

Item		Factor 1	Factor 2	Factor 3
**Ease of use and satisfaction (alpha=0.895), 8 items (MAUQ_E)**				
	I1. The app was easy to use.	*0.633*	–0.001	0.271
	I2. It was easy for me to learn to use the app.	0.234	–0.248	0.268
	I3. I like the interface of the app.	*0.729*	0.002	0.010
	I4. The information in the app was well organized, so I could easily find the information I needed.	*0.523*	–0.097	0.196
	I5. I feel comfortable using this app in social settings.	*0.538*	0.022	0.123
	I6. The amount of time involved in using this app has been fitting for me.	*0.588*	–0.041	0.222
	I7. I would use this app again.	*0.800*	–0.112	–0.136
	I8. Overall, I am satisfied with this app.	*0.855*	0.016	0.136
**System information arrangement (alpha=0.829), 6 items (MAUQ_S)**				
	I9. Whenever I made a mistake using the app, I could recover easily and quickly.	0.148	0.057	*0.672*
	I10. This mHealth app provided an acceptable way to receive health care services.	0.114	–0.189	*0.512*
	I11. The app adequately acknowledged and provided information to let me know the progress of my action.	0.281	0.007	*0.414*
	I12. The navigation was consistent when moving between screens.	0.178	–0.143	*0.466*
	I13. The interface of the app allowed me to use all the functions (such as entering information, responding to reminders, viewing information) offered by the app.	0.111	–0.129	*0.473*
	I14. This app has all the functions and capabilities I expect it to have.	0.180	–0.250	*0.485*
**Usefulness (alpha=0.900), 7 items (MAUQ_U)**				
	I15. The app would be useful for my health and well-being.	0.235	– *0.667*	–0.102
	I16. The app improved my access to health care services.	0.202	– *0.750*	–0.165
	I17. The app helped me manage my health effectively.	0.308	– *0.806*	–0.225
	I18. The app made it convenient for me to communicate with my health care provider.	–0.196	– *0.951*	0.109
	I19. Using the app, I had many more opportunities to interact with my health care provider.	–0.103	– *0.797*	0.146
	I20. I felt confident that any information I sent to my provider using the app would be received.	–0.046	– *0.541*	0.189
	I21. I felt comfortable communicating with my health care provider using the app.	–0.033	– *0.487*	0.257

The three factors correspond to three constructs, or subscales, on the MAUQ: ease of use and satisfaction (8 items, MAUQ_E), system information arrangement (6 items, MAUQ_S), and usefulness (7 items, MAUQ_U). Their Cronbach alpha values were 0.895, 0.829, and 0.900, respectively, which indicate strong internal consistency.

The correlation coefficients among the scores of the MAUQ, the scores of the MAUQ’s three subscales, the scores of the PSSUQ and its subscales, and the score of the SUS are shown in Table 3. The table shows that the three subscales in MAUQ are correlated. In addition, MAUQ_S is strongly correlated with PSSUQ1, which is related to system quality; MAUQ_E is strongly correlated with PSSUQ3, which is related to interface quality; MAUQ_U is correlated with the three subscales of the PSSUQ, but not as strongly as the other two MAUQ subscales. The reason is that MAUQ_U is mainly about the usefulness of the app for *health care*, which is not covered by the PSSUQ. For overall scores, the MAUQ is strongly correlated with the PSSUQ (*r*=0.8448) and also correlated with the SUS (*r*=0.6425). The correlation between the overall scores of the PSSUQ and the SUS is 0.6703. These correlation coefficient values show the criterion validity and construct validity of the MAUQ.

**Table 3:** Correlation coefficients among scores from the mHealth App Usability Questionnaire, Post-Study System Usability Questionnaire, System Usability Scale, and their subscales. Italics indicate strong correlations among the scales and their subscales.

Scales	MAUQ_U^a^	MAUQ_E^b^	MAUQ_S^c^	MAUQ^d^	PSSUQ1^e^	PSSUQ2^f^	PSSUQ3^g^	PSSUQ^h^
MAUQ_E	0.5775							
MAUQ_S	0.5745	0.7139						
MAUQ	0.8573	0.8794	0.8527					
PSSUQ1	0.5608	0.7657	*0.7124*	0.7781				
PSSUQ2	0.5194	0.6078	0.7044	0.6940	0.6466			
PSSUQ3	0.5129	*0.8077*	0.6474	0.7531	0.6857	0.5943		
PSSUQ	0.6078	0.8154	0.7957	*0.8448*	0.8815	0.8956	0.8267	
SUS^i^	0.4734	0.6367	0.5677	*0.6425*	0.6402	0.5238	0.6099	*0.6703*

^a^MAUQ_U: mHealth App Usability Questionnaire – usefulness.

^b^MAUQ_E: mHealth App Usability Questionnaire - ease of use and satisfaction.

^c^MAUQ_S: mHealth App Usability Questionnaire - system information arrangement.

^d^MAUQ: mHealth App Usability Questionnaire.

^e^PSSUQ1: Post-Study System Usability Questionnaire - subscale 1.

^f^PSSUQ2: Post-Study System Usability Questionnaire - subscale 2.

^g^PSSUQ3: Post-Study System Usability Questionnaire - subscale 3.

^h^PSSUQ: Post-Study System Usability Questionnaire.

^i^SUS: System Usability Scale.

#### mHealth App Usability Questionnaire for Standalone mHealth Apps (Patient Version)

For the MAUQ designed for standalone apps, a similar analysis was performed and again, three factors were found. We noticed that there were a few cross-loading items (overall satisfaction, information organization, usefulness, and time) when 0.32 was used as the cut-off value of factor loadings. We chose not to remove these items, since our experience and numerous other usability studies indicate the importance of measuring overall satisfaction, information organization, time spent on the app, and usefulness of the app. In the future, we will conduct studies with larger samples to further evaluate these statements and determine whether they should be kept in the standalone mHealth app usability evaluation.

The results for the EFA and correlation coefficient calculation are shown in Tables 4 and 5. Again, the values of Cronbach alpha in the overall questionnaire and the three subscales show strong internal consistency in the questionnaire. The correlation coefficients among MAUQ, PSSUQ, SUS, and their subscales also indicate the criterion validity and construct validity of the MAUQ.

**Table 4:** Exploratory factor analysis results for the 18 items on the mHealth App Usability Questionnaire designed for standalone mHealth apps (overall Cronbach alpha=0.914). Values >0.32 for each factor are italicized.

Item		Factor 1	Factor 2	Factor 3
**Ease of use (alpha=0.847), 5 items (MAUQ_E)**				
	S1. The app was easy to use.	*0.788*	0.121	–0.064
	S2. It was easy for me to learn to use the app.	*0.811*	0.090	–0.058
	S3. The navigation was consistent when moving between screens.	*0.708*	0.020	0.090
	S4. The interface of the app allowed me to use all the functions (such as entering information, responding to reminders, viewing information) offered by the app.	*0.640*	–0.055	0.271
	S5. Whenever I made a mistake using the app, I could recover easily and quickly.	*0.417*	–0.019	0.278
**Interface and satisfaction (alpha=0.908), 7 items (MAUQ_I)**				
	S6. I like the interface of the app.	0.223	*0.841*	–0.247
	S7. The information in the app was well organized, so I could easily find the information I needed.	0.525	*0.464*	–0.017
	S8. The app adequately acknowledged and provided information to let me know the progress of my action.	0.147	*0.450*	0.198
	S9. I feel comfortable using this app in social settings.	–0.020	*0.508*	0.186
	S10. The amount of time involved in using this app has been fitting for me.	0.321	*0.515*	0.092
	S11. I would use this app again.	-0.046	*0.680*	0.251
	S12. Overall, I am satisfied with this app.	0.323	*0.442*	0.301
**Usefulness (alpha=0.717), 6 items (MAUQ_U)**				
	S13. The app would be useful for my health and well-being.	–0.121	0.354	*0.584*
	S14. The app improved my access to health care services.	0.050	0.021	*0.390*
	S15. The app helped me manage my health effectively.	0.099	–0.002	*0.679*
	S16. This app has all the functions and capabilities I expected it to have.	0.210	0.178	*0.379*
	S17. I could use the app even when the Internet connection was poor or not available.	–0.014	0.025	*0.526*
	S18. This mHealth^a^ app provided an acceptable way to receive health care services, such as accessing educational materials, tracking my own activities, and performing self-assessment.	0.068	–0.016	*0.326*

^a^mHealth: mobile health.

**Table 5:** Correlation coefficients among scores from the mHealth App Usability Questionnaire, Post-Study System Usability Questionnaire, System Usability Scale, and their subscales. Italics indicate strong correlations among the scales and their subscales.

Scale	MAUQ_U^a^	MAUQ_E^b^	MAUQ_I^c^	MAUQ^d^	PSSUQ1^e^	PSSUQ2^f^	PSSUQ3^g^	PSSUQ^h^
MAUQ_E	0.5357							
MAUQ_I	0.5582	*0.7526*						
MAUQ	0.8053	0.8658	0.9122					
PSSUQ1	0.4684	0.8053	*0.8203*	0.8110				
PSSUQ2	0.5887	*0.7285*	0.7423	0.7961	0.8087			
PSSUQ3	0.5143	0.6411	0.7954	0.7654	0.7697	0.7604		
PSSUQ	0.5660	0.7979	0.8479	*0.8585*	0.9451	0.9373	0.8782	
iSUS	0.3832	0.7322	0.7362	*0.7168*	0.8513	0.7491	0.7476	*0.8523*

^a^MAUQ_U: mHealth App Usability Questionnaire - usefulness.

^b^MAUQ_E: mHealth App Usability Questionnaire - ease of use and satisfaction.

^c^MAUQ_I: mHealth App Usability Questionnaire - interface and satisfaction.

^d^MAUQ: mHealth App Usability Questionnaire.

^e^PSSUQ1: Post-Study System Usability Questionnaire - subscale 1.

^f^PSSUQ2: Post-Study System Usability Questionnaire - subscale 2.

^g^PSSUQ3: Post-Study System Usability Questionnaire - subscale 3.

^h^PSSUQ: Post-Study System Usability Questionnaire.

^i^SUS: System Usability Scale.

**Adjusted statement on the mHealth App Usability Questionnaire for use of mHealth apps by health care providers.**

Statements on the mHealth App Usability Questionnaire adjusted for interactive mobile apps designed for health care providers:

I10. This app provided an acceptable way to *deliver* health care services.

I15. The app would be useful for my health *care practice*.

I16. The app improved my access to *delivering* health care services.

I17. The app helped me manage my *patients’* health effectively.

I18. The app made it convenient for me to communicate with my *patients* **.**

I19. Using the app, I had many more opportunities to interact with my *patients*.

I20. I felt confident that any information I sent to my *patients* using the app will be received.

I21. I felt comfortable communicating with my *patients* using the app.

Statements on the mHealth App Usability Questionnaire adjusted for standalone apps designed for health care providers:

S13. The app would be useful for my *health care practice*.

S14. The app improved my access to *delivering* health care services.

S15. The app helped me manage my *patients’* health effectively.

S18. This mHealth app provided an acceptable way to *deliver* health care services, such as accessing educational materials, tracking my own activities, and performing self-assessment.

#### mHealth App Usability Questionnaire for Interactive and Standalone mHealth Apps (Provider Version)

In the versions of the MAUQ questionnaires described in the previous sections, some statements were generic, while some statements were specific to mHealth apps designed for patients. When evaluating the usability of mHealth apps designed for health care providers, some statements need to be slightly modified, for instance, changing the statement from *receiving* health care services to *delivering* health care services, and from interacting with *health care providers* to interacting with *patients*. Further evaluation may be needed for the health care provider version, since the two mHealth apps in this study were both for patients. Textbox 1 shows the statements on the MAUQ adjusted to evaluate mHealth apps designed for providers. The adjusted words are in italicized font.

### A Website for the mHealth App Usability Study Using the mHealth App Usability Questionnaire

To make it convenient for others to utilize the MAUQ in their mHealth app usability studies, we created a website [35] that includes the four versions of the MAUQ (interactive or standalone, patient or provider), and some optional demographic questions and open-ended questions typically used in usability studies. Anyone who wants to use this usability questionnaire can create an account on the website, create a customized usability questionnaire by simply selecting versions of the MAUQ and optional demographic questions, and add more demographic and open-ended questions if needed. The data collected in a usability study are stored on a secure Web server. The user can view a brief summary of the data collected in his/her usability study on the website and download the collected dataset to a local computer for further analysis.

## Discussion

### Principal Results

The aim of this study was to develop and validate a new mHealth app usability questionnaire—the MAUQ. This new questionnaire is highly reliable (reflected in the Cronbach alpha value and correlation with PSSUQ and SUS), as we used information gathered and summarized from many previous studies and combined the experience of several usability study experts to create it. Below is a summary of the unique contribution of our work.

First, the development of the MAUQ was based on a number of usability questionnaires used in mHealth app usability assessment described in published journal articles. Some of these questionnaires are well validated, and therefore, many statements in these questionnaires have been approved in numerous studies, making the use of a great resource a better choice than creation of a new questionnaire completely from scratch.

Second, the draft of the MAUQ was created considering components specific to mobile devices and mHealth apps. Some proposed mHealth app usability models were also consulted while questionnaire statements were being selected. This makes the MAUQ an mHealth app–specific questionnaire.

Third, a group of usability experts discussed the draft of the questionnaire and built the draft of the MAUQ. These usability experts had extensive experience in the study of mHealth app usability, and therefore, their knowledge and experience were integrated into the MAUQ during the discussion and questionnaire statement selection.

Fourth, four different versions of the MAUQ were created for four different scenarios (interactive app for patients, interactive app for health care providers, standalone app for patients, and standalone app for health care providers). This makes it feasible for others to easily choose one version for their usability study according to the desired context.

Fifth, this newly built questionnaire was tested in a usability study with 128 study participants and two mHealth apps—one, standalone (Fitbit) and the other, interactive (iMHere 2.0). This sample size is much bigger than typical usability studies; hence, it was feasible for an EFA. The data analysis results indicated that the MAUQ has strong construct validity and criterion validity, and the internal consistency of the three subscales and the entire questionnaire is high.

Sixth, the performance of the MAUQ was compared with two frequently used usability questionnaires—PSSUQ and SUS. The correlation coefficients among the MAUQ, PSSUQ, and SUS were high because they all were used to measure the overall usability of the mHealth apps. Two subscales of the MAUQ were also highly correlated with two subscales of the PSSUQ measuring similar aspects of usability. For the MAUQ subscale, with features unique to mHealth apps, the correlation was not that strong with any of the PSSUQ subscales, which is expected. In other words, the MAUQ can reliably measure usability of mHealth apps.

### Comparison With Prior Work

According to our knowledge, there is no single highly reliable usability questionnaire specifically designed for mHealth apps. Researchers from other teams have investigated usability models for mobile apps and also tried to modify existing usability questionnaires for use in mobile app usability studies. However, to date, none of these have been widely adopted by other researchers.

The 21 statements in the final version of the MAUQ for interactive mobile apps were compared with the ones in several other frequently used usability questionnaires. The result indicated that 5 statements in the MAUQ were highly similar to the ones in multiple other questionnaires; 10 statements in the MAUQ were similar to the ones in a few other questionnaires, but more specific to mobile app–based health care activities; and the remaining 6 statements were unique to the MAUQ.

A few years ago, a mobile app rating scale (MARS) was created for assessing the *quality* of mHealth apps [36], which is broader than usability. The target users of MARS were experts in one field (researchers, clinicians, and other professionals) who wanted to identify high-quality mobile apps in that field. Shortly thereafter, a simpler user version of MARS (uMARS) was created for the general population for the same purpose [37]. Both MARS and uMARS included some usability components such as engagement, functionality, and information quality; however, they were not specifically designed to study usability with end users.

One recently published work adjusted an existing usability questionnaire to apply it for mHealth apps [12]. In that study, the authors evaluated the psychometric properties of the existing usability questionnaire designed for health information technology systems by analyzing usability study data obtained from a group of patients with HIV. The authors indicated that this customizable health information technology usability questionnaire, the Health IT Usability Evaluation Scale (Health ITUES), worked well in an mHealth app usability study. However, it is tricky to customize the statements in a usability questionnaire, since the change may impact the responses of the study participants. In the original Health ITUES and the modified version for mHealth apps, several statements need to be customized by the questionnaire user [12,22]. Although customization makes it convenient to measure unique features offered by an individual health information technology system or mHealth app, it also creates challenges for questionnaire users, especially ones who are not experienced in questionnaire development, and this, in turn, will make the reliability and validity of the customized usability questionnaire questionable.

In the MAUQ, we provide four versions for two types of target users (patients and providers) and two major types of mHealth apps (standalone and interactive), which allows MAUQ users to choose the version that fits their needs. Moreover, the website created for the MAUQ makes the administration of the usability questionnaire easy. Researchers and mobile app developers can easily use the website to perform a quick usability/feasibility study with a small number of participants on the prototype of their apps, to conduct a multistage usability study during the process of the app development, or to have a large-scale intensive usability study with many participants over a long period of time. In addition, all the collected data from these usability studies are stored securely on the website for viewing and downloading.

### Limitations

The study was performed in the greater Pittsburgh area, but approximately one-third of the study participants were undergraduate and graduate students from different states, making the conclusions obtained in this study generalizable within certain limitations.

In this study, we had more female participants. All the participants were randomly selected from eligible candidates, but there were significantly more female mobile app users who expressed interest in this study. In other words, this study population was not an exact reflection of the US population; however, it did indicate the distribution of mobile app users who were interested in using mHealth apps to take care of their health.

The sample size (N=128) was considered sufficient for EFA, since the ratio between the number of study participants and the number of statements (n=21) was greater than 5. Since EFA is a large-sample procedure, a larger sample size provides more reliable results, for instance, to determine whether the items with low factor loading should be kept in the MAUQ for interactive apps and whether the cross-loading items should be removed from the MAUQ for standalone apps. For this reason, we will use this questionnaire in our usability studies to collect more data in the future. We also created a website to make it convenient for others to use this new questionnaire in their usability studies. We encourage others who use the MAUQ in their usability studies to share their collected data with us, so that we can perform further psychometric analysis on this questionnaire to improve its use for mHealth app usability evaluation.

In this study, we required that the study participants had certain characteristics, such as a high school or higher education, age between 18 and 65 years, and some experience using mHealth apps. This excluded some potential participants, for instance, people older than 65 years or people with a low education level. However, since the purpose of this study was to evaluate the newly created usability questionnaire, the participants selected were representative of the majority of mHealth app users and ones who could provide the most reliable assessment on the questionnaire. A different usability study method may be used for populations not included in this study.

The usability study in this work was performed on two mobile apps: iMHere 2.0 and Fitbit. It is possible that the results might have been *slightly* different for different mHealth apps. This can be assessed in the future after other research teams adopt the MAUQ in their usability studies with other mHealth apps.

Only the patient versions of the MAUQ were tested in this study. Therefore, although we believe the differences in the versions for patients and the versions for providers are not significant, the versions of MAUQ for health care providers were not explicitly evaluated. In the future, we may identify appropriate interactive and standalone mHealth apps designed for health care providers and perform the usability study again to evaluate the versions for providers. We may also utilize usability study data from other research teams to perform further assessment on the versions for providers.

## Abbreviations

CINAHL: Cumulative Index to Nursing & Allied Health Literature
EFA: exploratory factor analysis
Health ITUES: Health IT Usability Evaluation Scale
MARS: mobile app rating scale
MAUQ: mHealth App Usability Questionnaire
mHealth: mobile health
PSSUQ: Post-Study System Usability Questionnaire
SUS: System Usability Scale
